# Collaborative optimization of computational offloading and resource allocation based on Stackelberg game

**DOI:** 10.1371/journal.pone.0339955

**Published:** 2026-01-02

**Authors:** Lina Li, Qinghe Yu, Chundong Wang, Jiaqi Zhao, Junan Lv, Shuxin Wang, Chuncheng Hu

**Affiliations:** College of Computer Science and Technology, Changchun University, Changchun, China; Northwestern Polytechnical University, CHINA

## Abstract

The exponential growth of the Internet of Things and mobile edge computing has intensified the need for substantial data processing and instantaneous response. Consequently, collaboration between the cloud, the edge and the end has become a key computing paradigm. However, in this architecture, task scheduling is complex, resources are heterogeneous and dynamic, and it is still a serious challenge to achieve low-latency and energy-efficient task processing. Aiming at the deficiency of dynamic collaborative optimization in the existing research, this paper introduces a collaborative optimization approach for computational offloading and resource allocation, utilizing the Stackelberg game to maximize the system’s total utility. First, an overall utility model that integrates delay, energy consumption, and revenue is constructed for application scenarios involving multi-cloud servers, multi-edge servers, and multiple users. Subsequently, a three-tier Stackelberg game model is developed in which the cloud assumes the role of the leader, focusing on the establishment of resource pricing strategies. Concurrently, the edge operates as the sub-leader, fine-tuning the distribution of computational resources in alignment with the cloud’s strategic initiatives. Meanwhile, the mobile terminal functions as the follower, meticulously optimizing the computation offloading ratio in response to the superior strategies delineated by the preceding tiers. Next, through game equilibrium analysis, the existence and uniqueness of the Stackelberg equilibrium are proven. Finally, a BI-PRO is proposed based on the backward induction resource pricing, allocation, and computation offload optimization algorithm. The experimental findings indicate that the proposed Stackelberg game method optimizes the system’s total revenue and maintains stable performance across various scenarios. These results confirm the superiority and robustness of the method.

## Introduction

As the proliferation of mobile Internet and the Internet of Things continues, various terminal devices continue to generate massive data, and the demand for task processing is increasing. Cloud computing, recognized for its robust computational capabilities and expansive storage capacity, effectively addresses the challenges of large-scale task processing. However, it inherently faces a latency bottleneck, making it insufficient for the real-time demands of terminal devices. Edge computation relocates a portion of the computing and storage capacity to the user side, offering benefits such as reduced transmission delay and decreased load on cloud computing resources [1]. This approach is particularly suited for applications that require high real-time performance and are sensitive to delay. However, due to the limited computing and storage resources at the edge, it may not meet the demands of certain compute-intensive applications.

Addressing the aforementioned bottlenecks, collaboration between cloud, edge, and end can effectively allocate and utilize computing resources to cater to the diverse needs of large-scale applications. The terminal has the option to either process tasks locally or offload them to the edge or cloud for execution, thus leveraging the computational and storage capabilities of cloud computing as well as the low latency advantage of edge computation. For applications such as large-scale Internet of Things, smart cities, and intelligent transportation [2–4], this collaboration between cloud, edge, and end provides superior performance, reliability, flexibility, and high availability services.

In the cloud-edge-end scenario, the key of collaborative computing lies in determining the optimal resource allocation and offloading decisions. This is imperative to enhance computing efficiency, minimize latency, and optimize revenue. However, most of the works mainly consider the optimization from the perspective of resource allocation or computational offloading, and seldom involve the joint optimization of the two, and the optimization objective is relatively single. In addition, the existing research methods are mainly based on heuristic algorithms and reinforcement learning algorithms, which lead to the fact that the solution is not necessarily optimal, and there are problems such as training difficulties and slow convergence speed, and the optimal solution or approximate optimal solution is seldom explored. This paper thus sets its sights on the maximization of system utility within the cloud-edge-end framework. It takes into account factors such as time delay, energy consumption, and revenue to construct a three-level game model in game theory [5,6]. In this model, the primary leader focuses on reducing time delay while also aiming for higher revenue. The secondary leader, on the other hand, prioritizes reducing time delay alongside achieving better revenue and energy efficiency. This approach is intended to address the collaborative optimization problem of task offloading and resource allocation. The key contributions of this work are enumerated below.

(1) We introduce a three-tier Stackelberg game model aimed at optimizing the overall utility (encompassing delay, energy consumption, and revenue) within a cloud-edge-end collaborative environment. The cloud, serving as the primary leader, devises a delay-conscious resource pricing strategy. Subsequently, the edge adjusts its resource allocation in response to both revenue considerations and delay constraints. Finally, the terminal assesses and computes the offload ratio based on the resources that are available.

(2) We propose an optimization algorithm BI-PRO for resource pricing, allocation and computation offloading based on backward induction, which is used to solve the three-level Stackelberg game. Meanwhile, we optimize the efficiency of computational offloading in occlusion environment by improving Shannon’s formula, and dynamically select the offloading scheme.

(3) We prove the existence and uniqueness of the equilibrium solution for the three-layer Stackelberg game. Meanwhile, through simulation experiments, it is verified that BI-PRO has certain advantages and robustness. Compared with the full local computing method and random offloading method, this method can achieve higher total system utility in multiple different scenarios, and its performance is stable.

## Related work

In the cloud-edge-terminal framework, resource allocation and task offloading in mobile terminals remain key research focuses. In this section, we review studies related to cloud-edge collaboration and cloud-edge-terminal coordination, including traditional approaches and intelligent methods. Additionally, we employ a tabular comparison to analyze similarities and differences between our work and existing studies, while systematically examining limitations and challenges inherent in prior research as well as highlighting the innovative aspects of our approach.

### Traditional methods

Currently, the majority of research efforts are primarily focused on the cloud-edge collaborative computing paradigm and employ conventional methodologies. Typically, Chen and Xie et al. [7,8] aimed to minimize latency and reduce energy consumption of terminal devices by decomposing a mixed integer programming optimization problem into two or three convex optimization subproblems, and obtained the optimal solution using KKT conditions and alternating optimization techniques. Zhang and Wang et al. [9,10] aimed to minimize energy consumption (both network and end users), considering task delay constraints or optimization, and employed methods based on bipartite graph matching to design low-complexity edge-cloud matching algorithms. Wang et al. [11] proposed a cloud-edge transaction collaboration framework and designed an optimal resource matching transaction scheme to maximize MEC profit while ensuring delay requirements. Li and Truong et al. [12,13] focused on the joint optimization of computation offloading and resource allocation in MEC, and proposed solutions based on intelligent methods such as genetic algorithms to optimize device energy consumption or task latency. Alqahtani et al. [14] proposed a greedy heuristic allocation algorithm based on multi-dimensional factors including task deadlines, node locations, and budget constraints to address the optimization problem of task allocation between edge and cloud resources. Zhu et al. [15] developed a collaborative task scheduling mechanism integrating cloud and edge computing environments, and introduced a cooperative computation-based task scheduling algorithm that achieved improved computational efficiency. Chen et al. [16] proposed a three-tier collaborative offloading framework based on the Lyapunov optimization method, achieving the objective of minimizing the system’s total energy consumption. Long et al. [17] aiming to minimize the total energy consumption of mobile devices, introduced an edge-cloud rental collaboration architecture along with greedy and simulated annealing algorithms. Ren et al. [18] established a joint optimization model for communication and computational resource allocation, employing convex optimization theory and the Lagrange multiplier method to solve for optimal efficiency in both communication and computational resources.

### Intelligent methods

In recent years, research efforts based on intelligent methods have also achieved preliminary progress. Typically, Wang et al. [19] established a task execution model termed “mobile terminal-edge cloud-remote cloud” and proposed a weighted adaptive inertia weight particle swarm optimization algorithm (WAIW-PSO) based on multi-cloud collaboration to address the challenges of task offloading and resource contention. Wang et al. [20] proposed a task offloading strategy based on particle swarm optimization and genetic algorithm to address the task offloading problem in device-edge-cloud collaborative computing (DE3C) systems, demonstrating superior performance in terms of task acceptance rate and resource utilization efficiency. Tang et al. [21] constructed a new paradigm of cloud-edge collaborative computing unloading by deep integration of satellite network and ground edge computation resources, and employed a hybrid solution framework that synergizes a deep neural network with a successive convex approximation algorithm. Sun et al. [22] introduced a sophisticated service deployment approach tailored for delay-sensitive Internet of Things (IoT) service optimization, which hinges on event log timing constraints and employs an enhanced non-dominated sorting genetic algorithm for resolution. Ning et al. [23] proposed a computation offloading and task scheduling algorithm for vehicular networks, which employs convex optimization to determine the offloading ratios of vehicle users, and constructs a non-cooperative game model to achieve maximization of the overall benefits of both users and network operators. Hao et al. [24] established a Markov decision process model with the objective of maximizing the long-term average delay reduction for the cloud edge service offloading problem, and employed a multi-update reinforcement learning algorithm to reduce the scale of the action space. Gottam et al. [25] proposed a human evolution-assisted deep reinforcement learning model (HEOp-DRL) to address device-assisted MEC task offloading and resource allocation, aiming to minimize overall latency and energy consumption. Zhang et al. [26] proposed a privacy-preserving task offloading scheme based on Deep Q-Network (DQN) for edge-cloud collaboration (ECC) scenarios to reduce latency and energy consumption while enhancing privacy preservation.

We compare our main contributions with existing representative works in Table 1. In existing distributed service systems, there exist complex interdependencies and conflicts among resource allocation, service pricing, and user demands. As shown in Table 1, traditional approaches primarily focus on bipartite models or single-objective optimizations, which fail to capture such multi-level, multi-objective coordination challenges. We employ game theory to incorporate platforms, service providers, and users into a unified analytical framework, simultaneously optimizing three key metrics: latency, energy consumption, and revenue. To address the computational challenges of solving this complex game, we design and theoretically validate an efficient backward induction algorithm that not only guarantees finding system equilibrium points under quality-of-service constraints but also demonstrates low computational complexity.

**Table 1:** Comparison of our work with the most advanced intelligent methods.

Model	Layers	Latency	Energy	Rewards	Methods
WAIW-PSO [19]	terminal-cloud	✓	✗	✗	PSO
PSO-GA [20]	device–edge–cloud	✓	✗	✗	PSO and GA
DNN-SCA [21]	device–edge–cloud	✓	✓	✗	DNN and SCA
NDSGA [22]	edge–cloud	✓	✓	✗	GA
POETS [23]	device–edge	✗	✗	✓	Game theory
MUDRL [24]	edge–cloud	✓	✗	✗	DRL
HEOp–DRL [25]	device–device–edge	✓	✓	✗	EA and DRL
PP-DQN [26]	device–edge–cloud	✓	✓	✗	DRL
Our work	device–edge–cloud	✓	✓	✓	Game theory

### Challenges and our approach

In summary, current research primarily focuses on end-side architectures or edge-cloud architectures, while the joint optimization of cloud-edge collaborative architectures remains insufficiently explored, which constrains the overall system performance. Meanwhile, the complexity of task allocation and the dynamic nature of heterogeneous resources across cloud-edge-end continuum result in persistent challenges of high latency and energy consumption in task offloading (computation offloading). This paper aims to achieve joint optimization of energy consumption, latency, and revenue objectives under cloud-based architectures. We construct a three-layer Stackelberg game model that captures complex interdependencies and conflicts among resource allocation, service pricing, and user demands. An efficient backward induction solution algorithm is designed to guarantee optimal system utility under complex constraints through mechanism design. Our work mechanistically reveals that hierarchical pricing and strategic guidance can enable system-level collaborative optimization, providing theoretical foundations and design guidelines for developing next-generation service systems characterized by high profitability, operational efficiency, and low energy consumption.

## System model based on Stackelberg game

**Fig 1:**
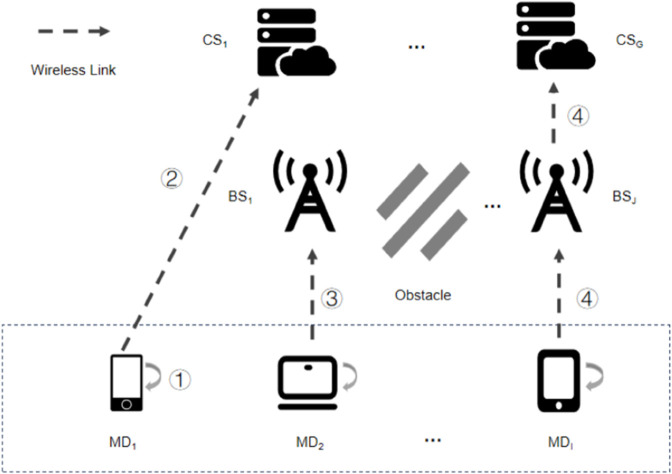
Framework of cloud-edge-end collaboration system.

This study examines a three-tier mobile edge computing (MEC) system, which comprises *G* Cloud Servers (CS), *J* Base Stations (BS), and *I* Mobile Devices (MD) positioned at the cloud-edge-end spectrum. The system framework is shown in Fig 1. All servers and end devices are outfitted with antennas and are arranged in an Orthogonal Frequency Division Multiple Access (OFDMA) configuration [27,28] to facilitate wireless communication between mobile devices and servers. Remote clouds have more computing resources, but have the highest communication costs. Each base station deploys edge servers with limited computing power, closer to the user than remote cloud [29]. Every mobile device is tasked with gathering various user tasks. However, these devices have a limited range of resources, which leads to task offloading. This offloading process can be divided into four specific scenarios. In the first scenario, the computing tasks of mobile device *MD*_1_ are processed locally (depicted as 1). In the second scenario, *MD*_1_ offloads a portion of its computing tasks to cloud server *CS*_1_ for processing (represented as 2). In the third scenario, mobile device *MD*_2_ delegates a part of its computing tasks to the edge server of base station *BS*_1_ for processing (illustrated as 3). In the fourth and final scenario, when an obstacle obstructs the transmission signals between mobile device *MD*_1_ and cloud server *CS*_*G*_, the computing task is routed to base station *BS*_*J*_ to serve as a relay node, and subsequently forwarded to cloud server *CS*_*G*_ for processing (portrayed as 4).

We define the total quantity of mobile devices as *I*, with the *i*-th mobile device represented as *MD*_*i*_, 1≤i≤I. The $T_{i}≜(D_{i},\varphi_{i})$ represents a computationally intensive task on *MD*_*i*_. *D*_*i*_ represents the amount of computation of task *T*_*i*_. The quantity $\varphi_{i}$ represents the number of CPU cycles necessary for the processing of task *T*_*i*_. The ratio vector for the calculation performed locally by the mobile device is $\lambda^{l}=\left[{\lambda_{1}}^{l},{\lambda_{2}}^{l},\cdots ,{\lambda_{I}}^{l}\right]$. The mobile device will compute the offload ratio vector to the edge server *ES*_*j*_ as $\lambda^{j}=\left[{\lambda_{1}}^{j},{\lambda_{2}}^{j},\cdots ,{\lambda_{I}}^{j}\right]$. The mobile device will calculate the offload ratio vector to the cloud server *CS*_*g*_ as $\lambda^{g}=\left[{\lambda_{1}}^{g},{\lambda_{2}}^{g},\cdots ,{\lambda_{I}}^{g}\right]$. Further, the mobile device will calculate the offload ratio vector $\lambda^{r}=\left[{\lambda_{1}}^{r},{\lambda_{2}}^{r},\cdots ,{\lambda_{I}}^{r}\right]$ of offloading to the cloud server via edge server. Within this framework, users are required to assume the computational overhead associated with task handover to the processing system. This typically includes considerations of time cost, energy consumption, and monetary expenditure. The cloud and edge servers are presumed to be equipped with high-performance multi-core CPUs. Consequently, their computing energy consumption is deemed negligible in comparison to the communication energy consumption, which is the focus of this study. The objective of this paper is to optimize the total utility of cloud-edge-end systems in executing tasks for *I* mobile devices. Specifically, the aim is to maximize user satisfaction through computation offloading, while minimizing system costs. For the convenience of readers, we have summarized the main relevant symbols in Table 2.

**Table 2:** Definitions of main notations.

Notation	Description
${B_{i}}^{j}$	The bandwidth between terminal devices and edge servers
${B_{i}}^{g}$	The bandwidth between terminal devices and cloud servers
${B_{j}}^{g}$	The bandwidth between edge servers and cloud servers
${P_{i}}^{j}$	The transmission power of the uplink from terminal devices to edge servers
${P_{i}}^{g}$	The transmission power of the uplink from terminal devices to cloud servers
${P_{j}}^{g}$	The transmission power of the uplink from edge servers to cloud servers
$\sigma^{2}$	Additive white Gaussian noise power
$\eta_{0}$	Channel power gain constant
*k*_*j*_	Effective switched capacitance of edge servers
*k*_*l*_	Effective switched capacitance of terminal devices
*N*_*NLos*_	Interference power from obstacles between terminal devices and cloud servers
*F* ^*j*^	The computation frequency of edge servers
*F* ^*g*^	The computation frequency of cloud servers
${M_{i}}^{g}$	The price of resource usage by mobile devices on cloud servers
${M_{j}}^{g}$	The price of resource usage on cloud servers by edge servers
${m_{i}}^{j}$	The price of resource usage by mobile devices on edge servers
$Rol{e_{i}}^{j}$	The role played by edge servers
*D*_*i*_	The computation amount of task *i*
$\varphi_{i}$	The number of CPU cycles
${\tau_{i}}^{g}$	The obstacle interference between terminal devices and cloud servers

### Cloud master leader model

In the three-tier Stackelberg non-cooperative master-slave game spanning cloud, edge, and end layers, the cloud server exhibits the most robust processing capability and functions as the leader in the first sub-game layer. The cloud server, designated *CS*_*g*_, is tasked with establishing the price ${M_{i}}^{g}$ for the computing resources sought by each mobile device, labeled *MD*_*i*_, as well as the price *m* for the relay edge server utilized, designated *ES*_*j*_. The available computation frequency of the cloud server is represented as ${M_{j}}^{g}$, while the computation frequency allocated to the mobile device *MD*_*i*_ is denoted as ${F_{i}}^{g}$. The cloud server’s profit from offering computing services to the terminal device is expressed as ${P_{g}}^{comp}$, and the cost associated with employing the edge server is indicated as ${C_{g}}^{r}$. The processing time for task *T*_*i*_ within the cloud encompasses both the computation time, ${T_{i,g}}^{comp}$, and the communication time, ${T_{i,g}}^{com}$. However, from the cloud’s perspective, the allocation of computing resources is of primary concern, rendering the communication time effectively negligible. The variable *U*_*g*_ characterizes the total utility of the cloud server, defined as the arithmetic difference between the overall benefit and the aggregate completion time. Every cloud server *CS*_*g*_ endeavors to optimize its total revenue from processed tasks while simultaneously minimizing the overall task completion time. Therefore, the optimization index of the cloud at the leader level is formally expressed as

$${P_{g}}^{comp}=\sum_{i\in I}M_{i}^{g}F_{i}^{g}S_{i}^{g},$$ (1)

$${C_{g}}^{r}=\sum_{i\in I}\sum_{j\in J}{M_{j}}^{g}D_{i}{\lambda_{i}}^{r}{S_{i}}^{j},$$ (2)

$${T_{i,g}}^{comp}=\frac{({\lambda_{i}}^{g}+{\lambda_{i}}^{r})\varphi_{i}}{{F_{i}}^{g}},$$ (3)

$$U_{g}=\gamma^{p}({P_{g}}^{comp}-{C_{g}}^{r})-\gamma^{t}{T_{i,g}}^{comp}.$$ (4)

In the equations provided, the variable ${S_{i}}^{g}=1$ indicates that mobile device *MD*_*i*_ offloads its computational task to cloud server *CS*_*g*_, while ${S_{i}}^{g}=0$ denotes the opposite. Similarly, ${S_{i}}^{j}=1$ suggests that the computational task of mobile device *MD*_*i*_ requires edge side assistance, and conversely, ${S_{i}}^{j}=0$ indicates otherwise. The variable ${\lambda_{\text{i}}}^{\text{g}}$ quantifies the proportion of the computational task of mobile device *MD*_*i*_ offloaded to the cloud server. Meanwhile, ${\lambda_{i}}^{r}$ quantifies the proportion of the computational task of *MD*_*i*_ offloaded to the edge server *ES*_*j*_ for relay to the cloud server. The variable $\varphi_{\text{i}}$ records the number of cycles needed for task processing. Furthermore, $\gamma^{\text{p}}$ and $\gamma^{\text{t}}$ represent the weight of payoff and time, respectively, both contributing to the utility of the terminal device such that their sum equals 1.

Thus, the first layer of the positive dominant subgame is expressed as

$$\max U_{g}({M_{i}}^{g},{M_{j}}^{g},{F_{i}}^{g}),\forall i\in I,j\in J,\;s.t.\;C1:\sum_{i=1}^{I}{F_{i}}^{g}\leq F^{g},\;\;C2:{M_{i}}^{g}\in \left[\min{M_{i}}^{g},\max{M_{i}}^{g}\right],\;\;C3:{M_{j}}^{g}\in \left[\min{M_{j}}^{g},\max{M_{j}}^{g}\right],\;\;C4:{\text{j}}_{g}⋂j_{g^{\prime }}=\emptyset ,\forall g,g^{\prime }\in G,\;$$ (5)

where ${M_{i}}^{g}$, ${M_{j}}^{g}$ and ${F_{i}}^{g}$ represent the variable parameters of resource pricing and resource allocation that need to be solved by the subsequent algorithm. The total available computing frequency of the could server is represented by *F*^*g*^. Constraint *C*1 dictates that the computational resources of the cloud server are finite. Constraints *C*2 and *C*3 stipulate that the costs associated with offering computing offload service customization for mobile devices and the expenses related to the employment of edge servers should remain within specified bounds. Furthermore, Constraint *C*4 states that each edge server may only be utilized by a single cloud server as a relay [30].

### Edge-end vice-leader model

Similarly, in the three-level Stackelberg non-cooperative master-slave game at the cloud-edge-end, the edge server has strong processing power and plays the role of the deputy leader, which is in the second-level sub-game. Based on the free space loss model, we construct the data transmission model between the layers. The data transfer rates between the terminal device and the cloud server, the terminal device and the edge server, and the edge server and the cloud server are denoted as

$${R_{i}}^{g}=B_{i}^{g}\log_{2}(1+\frac{P_{i}^{g}\eta_{0}{\left({d_{i}}^{g}\right)}^{-2}}{\sigma^{2}+{\tau_{i}}^{g}N_{NLos}}),$$ (6)

$${R_{i}}^{j}=B_{i}^{j}\log_{2}(1+\frac{P_{i}^{j}\eta_{0}{\left({d_{i}}^{j}\right)}^{-2}}{\sigma^{2}}),$$ (7)

$${R_{j}}^{g}=B_{j}^{g}\log_{2}(1+\frac{P_{j}^{g}\eta_{0}{\left({d_{j}}^{g}\right)}^{-2}}{\sigma^{2}}),$$ (8)

where $B_{i}^{g}$ denotes the bandwidth between terminal devices and the cloud server, $B_{i}^{j}$ represents the bandwidth between terminal devices and the edge server, $B_{j}^{g}$ indicates the bandwidth between the edge server and the cloud server, $P_{i}^{g}$ signifies the transmission power for tasks uploaded from terminal devices to the cloud server, $P_{i}^{j}$ refers to the transmission power for tasks uploaded from terminal devices to the edge server, and $P_{j}^{g}$ corresponds to the transmission power for tasks uploaded from the edge server to the cloud server. The channel power gain constant among the terminal device, the edge server, and the cloud server is represented by $\eta_{0}$ [31]. The physical distances between the terminal device and the cloud server, between the terminal device and the edge server, and between the edge server and the cloud server are denoted as ${d_{i}}^{g}$, ${d_{i}}^{j}$ and ${d_{j}}^{g}$ respectively. The path loss from the terminal device to the cloud server, from the terminal device to the edge server, and from the edge server to the cloud server are represented as ${\left({d_{i}}^{g}\right)}^{-2}$, ${\left({d_{i}}^{j}\right)}^{-2}$ and ${\left({d_{j}}^{g}\right)}^{-2}$ respectively. $\sigma^{2}$ is the additive white Gaussian noise power of the transmission channel [32]. The interference power of an obstacle situated between the terminal device and the cloud server is denoted as *N*_*NLos*_. If there is an obstacle between the terminal device and the cloud server, then ${\tau_{i}}^{g}=1$ represents its interference; otherwise, the interference is defined as ${\tau_{i}}^{g}=0$.

The aforementioned transmission rates, represented as ${R_{i}}^{g}$, ${R_{i}}^{j}$, and ${R_{j}}^{g}$, facilitate the edge server’s decision-making process regarding whether to directly address the computational task of the terminal device. Here, the edge server’s decision corresponds to

$$Rol{e_{i}}^{j}=\{\begin{array}{l} 0,{R_{i}}^{g}<\frac{{R_{i}}^{j}+{R_{j}}^{g}}{2}\\ 1,{R_{i}}^{g}\geq \frac{{R_{i}}^{j}+{R_{j}}^{g}}{2} \end{array} .$$ (9)

In instances where the transmission rate between the terminal device and the cloud server equals or exceeds the average transfer rate of the edge server, the edge server directly executes the calculation task (${\delta_{j}}^{g}=1$) for the terminal device. Conversely, when the transmission rate is lower than the edge server’s average transfer rate, the edge server serves as a relay node, forwarding the calculation task (${\delta_{j}}^{g}=0$) from the terminal device.

Further, ${P_{j}}^{comp}$ and ${P_{j}}^{relay}$ denote the profits of the edge server when functioning as a computing node and a relay node, respectively. The variable ${m_{i}}^{j}$ signifies the cost of the computing resource utilized by the mobile device *MD*_*i*_. The total available computing frequency of the edge server is represented by *F*^*j*^, while ${F_{i}}^{j}$ indicates the CPU frequency specifically allocated to the mobile device *MD*_*i*_. Consequently, the profits of the deputy leader, ${P_{j}}^{comp}$ and ${P_{j}}^{relay}$, can be expressed as

$${P_{j}}^{comp}=\sum_{i\in I}{m_{i}}^{j}{F_{i}}^{j}Rol{e_{i}}^{j},$$ (10)

$${P_{j}}^{relay}=\sum_{i\in I}\sum_{i\in J}{M_{j}}^{g}D_{i}{\lambda_{i}}^{r}{S_{i}}^{j}(1-Rol{e_{i}}^{j}),$$ (11)

where $Rol{e_{i}}^{j}=1$ indicates that the edge end *ES*_*j*_ acts as a computing node to handle the task of the mobile device *MD*_*i*_, otherwise $Rol{e_{i}}^{j}=0$, acts as a relay node.

When the edge end *ES*_*j*_ plays the role of a compute node, there is a computational overhead ${E_{j}}^{comp}$ to process the task, that is

$${E_{j}}^{comp}=\sum_{i\in I}k_{j}{\left({F_{i}}^{j}\right)}^{2}\varphi_{i}{\lambda_{i}}^{j}Rol{e_{i}}^{j},$$ (12)

where *k*_*j*_ represents the effective switched capacitance of the edge server, which is determined by the chip architecture and is typically set to 10^−27^.

In the process of task transmission, the edge end *ES*_*j*_ also incurs a communication cost, defined as

$${E_{j}}^{comm}=\sum_{i\in I}{\sum_{i\in J}P_{j}}^{g}\frac{D_{i}}{{R_{j}}^{g}}{\lambda_{i}}^{r}(1-Rol{e_{i}}^{j}).$$ (13)

The processing duration for task *T*_*i*_ at the edge end is comprised of both the computation time, denoted as ${T_{i,j}}^{comp}$, and the communication time, represented as ${T_{i,j}}^{comm}$. Since the edge side is concerned with the allocation of computational resources, the transmission time portion is negligible. The computation time ${T_{i,j}}^{comp}$ is formally expressed as

$${T_{i,j}}^{comp}=\frac{{\lambda_{i}}^{j}\varphi_{i}}{{F_{i}}^{j}}.$$ (14)

The objective of each edge server, denoted as *ES*_*j*_, is twofold: to maximize the revenue derived from processed tasks and to minimize their total completion time. The variable *U*_*j*_ quantifies the total utility of the edge server, defined as the difference between its total revenue and total task completion time. The coefficients $\beta^{p}$, $\beta^{e}$, and $\beta^{t}$ weight the contributions of profit, energy consumption, and time, respectively, to the utility of the edge device. Notably, these weights sum to 1, and *U*_*j*_ is given by

$$U_{j}=\beta^{p}({P_{j}}^{comp}+{P_{j}}^{relay})-\beta^{e}({E_{j}}^{comp}+{E_{j}}^{comm})-\beta^{t}{T_{i,j}}^{comp}.$$ (15)

Therefore, the second-level vice-dominant subgame can be expressed as

$$\max U_{j}({m_{i}}^{j},{F_{i}}^{j}),\forall i\in I,\;s.t.\;C5:\sum_{i=1}^{I}{F_{i}}^{j}\leq F^{j},\;\;C6:\;{m_{i}}^{j}\in \left[\min{m_{i}}^{j},\max{m_{i}}^{j}\right],\;\;C7:Rol{e_{i}}^{j}\in \left\{0,1\right\},\;$$ (16)

where ${m_{i}}^{j}$ and ${F_{i}}^{j}$ represent the variable parameters of resource pricing and resource allocation that need to be solved by the subsequent algorithm. Constraint *C*5 dictates that the computational resources of the edge servers are finite. Constraint *C*6 indicates that the price at which the edge server provides the computation offload service customization for the mobile device is within a certain range. Constraint *C*7 indicates that the edge server can act as either a compute node or a relay node.

### Terminal follower model

In the three-tier Stackelberg non-cooperative master-slave game across the cloud-edge-end spectrum, the terminal device serves as a follower, occupying the third tier of the sub-game. Its position is contingent upon the resource pricing and the availability of resources from both the cloud and edge servers to ascertain the task offloading ratio. As a follower, the mobile device *MD*_*i*_ takes into account the payment and processing costs of the computing service (including processing latency and processing energy consumption in transmission).

When a task is processed locally, the associated costs include the payment cost, denoted as ${C_{i}}^{l}$, the processing delay, represented as ${T_{i}}^{l}$, and the energy consumption for processing, which is ${E_{i}}^{l}$. We formally define them as follows:

$${C_{i}}^{l}=0,$$ (17)

$${T_{i}}^{l}=\frac{\varphi_{i}}{{F_{i}}^{j}},$$ (18)

$${E_{i}}^{l}=k_{l}{\left({F_{i}}^{j}\right)}^{2}\varphi_{l},$$ (19)

where *k*_*l*_ represents the effective switched capacitance of the mobile device, which is determined by the chip architecture and set to 10^−26^ based on practical configurations.

In instances where tasks are directly transitioned from a mobile device to the cloud, there are associated costs. These include the payment cost, denoted as ${C_{i}}^{g}$, the processing delay, represented by ${T_{i}}^{g}$, and the energy consumption during processing, symbolized as ${E_{i}}^{g}$. These notation are defined as

$${C_{i}}^{g}={M_{i}}^{g}{F_{i}}^{g},$$ (20)

$${T_{i}}^{g}=\frac{D_{i}}{{R_{i}}^{g}}+\frac{\varphi_{i}}{{F_{i}}^{g}},$$ (21)

$${E_{i}}^{g}={P_{i}}^{g}\frac{D_{i}}{{R_{i}}^{g}}.$$ (22)

When a task is offloaded from a mobile device to the edge end, three factors come into play: the payment cost, denoted as ${C_{i}}^{j}$ , the processing delay, represented as ${T_{i}}^{j}$ , and the processing energy consumption, symbolized as ${E_{i}}^{j}$. These variables must be considered in any analysis, and can be expressed as

$${C_{i}}^{j}={m_{i}}^{j}{F_{i}}^{j},$$ (23)

$${T_{i}}^{j}=\frac{D_{i}}{{R_{i}}^{j}}+\frac{\varphi_{i}}{{F_{i}}^{j}},$$ (24)

$${E_{i}}^{j}={P_{i}}^{j}\frac{D_{i}}{{R_{i}}^{j}}.$$ (25)

When the task is offloaded to the cloud via an edge end acting as a relay node, there is an associated cost denoted as ${C_{i}}^{r}$. Additionally, we can observe the processing delay, represented by ${T_{i}}^{r}$, and the energy consumption for processing, which is denoted as ${E_{i}}^{r}$. Specifically, the definitions of cost and delay are as follows:

$${C_{i}}^{r}={M_{i}}^{g}{F_{i}}^{g},$$ (26)

$${T_{i}}^{r}=\frac{D_{i}}{{R_{i}}^{j}}+\frac{\varphi_{i}}{{F_{i}}^{g}}+\frac{D_{i}}{{R_{j}}^{g}},$$ (27)

$${E_{i}}^{r}={P_{i}}^{j}\frac{D_{i}}{{R_{i}}^{j}}+{P_{j}}^{g}\frac{D_{i}}{{R_{j}}^{g}}.$$ (28)

In summary, the total cost *C*_*i*_ associated with the payment fee, processing delay, and energy consumption of the follower terminal device can be articulated as

$$C_{i}=\left\{ \begin{array}{cc} {\alpha_{l}}^{time}{T_{i}}^{l}+{\alpha_{l}}^{energy}{E_{i}}^{l}, & \lambda_{i}^{l}>0,\\ {\alpha_{ig}}^{pay}{C_{i}}^{g}+{\alpha_{ig}}^{time}{T_{i}}^{g}+{\alpha_{ig}}^{energy}{E_{i}}^{g}, & \lambda_{i}^{g}>0,\\ {\alpha_{ij}}^{pay}{C_{i}}^{j}+{\alpha_{ij}}^{time}{T_{i}}^{j}+{\alpha_{ij}}^{energy}{E_{i}}^{j}, & \lambda_{i}^{j}>0,\\ {\alpha_{r}}^{pay}{C_{i}}^{r}+{\alpha_{r}}^{time}{T_{i}}^{r}+{\alpha_{r}}^{energy}{E_{i}}^{r}, & \lambda_{i}^{r}>0, \end{array}\right.$$ (29)

where ${\alpha_{l}}^{time}$ and ${\alpha_{l}}^{energy}$ are the weights associated with processing delay and energy consumption for tasks processed locally. Conversely, ${\alpha_{ig}}^{pay}$, ${\alpha_{ig}}^{time}$, and ${\alpha_{ig}}^{energy}$ are the weights that pertain to the payment, processing delay, and energy consumption for tasks offloaded directly from the mobile device to the cloud. In a similar vein, ${\alpha_{ij}}^{pay}$, ${\alpha_{ij}}^{time}$ and ${\alpha_{ij}}^{energy}$ are the weights associated with the costs of payment, processing delay, and energy consumption for tasks offloaded from the mobile device to the edge server. Furthermore, ${\alpha_{r}}^{pay}$, ${\alpha_{r}}^{time}$ and ${\alpha_{r}}^{energy}$ represent the weight of the payment, processing delay, and processing energy consumption for tasks offloaded to the cloud via an edge end relay node. Finally, ${\lambda_{i}}^{l}$ represents the proportion of tasks executed locally, while ${\lambda_{i}}^{g}$, ${\lambda_{i}}^{j}$, and ${\lambda_{i}}^{r}$ respectively denote the proportions of tasks offloaded to the cloud server, edge server, and tasks offloaded to the cloud server through an edge server.

In the process of task offloading, the terminal device’s objective is to achieve the shortest task time while minimizing cost and energy consumption. The utility of the terminal device, denoted as *U*_*i*_, is defined as the negative sum of the payment fee, processing time, and processing energy consumption, thus $U_{i}=-C_{i}$. Consequently, the third-level subordinated subgame can be articulated as

$$\max U_{i}({\lambda_{i}}^{l},{\lambda_{i}}^{g},{\lambda_{i}}^{j},{\lambda_{i}}^{r}),\;s.t.C8:{\lambda_{i}}^{l},{\lambda_{i}}^{g},{\lambda_{i}}^{j},{\lambda_{i}}^{r}\in \left[0,1\right],\;\;C9:{\lambda_{i}}^{l}+{\lambda_{i}}^{g}+{\lambda_{i}}^{j}+{\lambda_{i}}^{r}=1.\;$$ (30)

Constraints *C*8 and *C*9 stipulate that the terminal device’s task can be partially executed on the local, edge, and cloud servers, with the total execution ratios summing up to 1.

### Cloud-edge-end computing offload model

Based on the above game sub-model, the cloud server, edge server, and mobile device are analogous to the primary leader, secondary leader, and follower respectively. This establishes a three-tier Stackelberg non-cooperative two-leader one-follower model for computing offloading. This model is formally articulated as

$$\psi =\left\{\left(G,J,I\right),\left(𝔾,𝕁,𝕀\right),\left(U_{g},U_{j},U_{i}\right)\right\},$$ (31)

where 𝔾, 𝕁, and 𝕀 represent the strategy spaces for the cloud server, edge server, and mobile device, respectively. For the three-layer Stackelberg non-cooperative master-slave game model with two masters and one slave, to obtain the equilibrium solution of the master-slave game, the set of key parameters $\left({M_{i}}^{g*},{M_{j}}^{g*},{m_{i}}^{j*},{F_{i}}^{g*},{F_{i}}^{j*},{\lambda_{i}}^{l*},{\lambda_{i}}^{g*},{\lambda_{i}}^{j*},{\lambda_{i}}^{r*}\right)$ of the three-layer sub-game that reach the equilibrium state needs to meet the following conditions, i.e.,

$$U_{g}\left({M_{i}}^{g*},{M_{j}}^{g*},{F_{i}}^{g*},{𝕁}^{*},{𝕀}^{*}\right)\geq U_{g}\left({M_{i}}^{g},{M_{j}}^{g},{F_{i}}^{g},{𝕁}^{*},{𝕀}^{*}\right),$$ (32)

$$U_{j}\left({𝔾}^{*},{m_{i}}^{j*},{F_{i}}^{j*},{𝕀}^{*}\right)\geq U_{j}\left({𝔾}^{*},{m_{i}}^{j},{F_{i}}^{j},{𝕀}^{*}\right),$$ (33)

$$U_{i}\left({𝔾}^{*},{𝕁}^{*},{\lambda_{i}}^{l*},{\lambda_{i}}^{g*},{\lambda_{i}}^{j*},{\lambda_{i}}^{r*}\right)\geq U_{i}\left({𝔾}^{*},{𝕁}^{*},{\lambda_{i}}^{l},{\lambda_{i}}^{g},{\lambda_{i}}^{j},{\lambda_{i}}^{r}\right).$$ (34)

In this paper, we utilize the total utility of the system, which is derived from the utilities of a three-level game, as a metric for evaluating the performance of the algorithm unloading process. The total utility of the system is defined as

$$U=U_{g}+U_{j}+U_{i}.$$ (35)

## Analysis of Stackelberg game equilibrium solution

### Proof of existence of equilibrium solution

Theorem 1. In the three-layer Stackelberg game model at the cloud edge, the equilibrium solution exists.

Proof: Given that the binary constraint $Rol{e_{i}}^{j}\in \left\{0,1\right\}$ (C7) leads to non-convexity of the edge server policy space, we first construct a relaxed problem. Specifically, we relax the constraint C7 to $Rol{e_{i}}^{j}\in \left[0,1\right]$, allowing the edge servers to mix their role policies with a certain probability.

After this relaxation, the strategy set 𝔾,𝕁,𝕀 of the cloud, edge and terminal parties is non-empty, compact and convex. It can be shown that, given the strategies of the other players, the optimal response map for each player is upper semicontinuous. According to Kakutani’s fixed point theorem [33], there is an equilibrium point $\left({M_{i}}^{g*},{M_{j}}^{g*},{m_{i}}^{j*},{F_{i}}^{g*},{F_{i}}^{j*},{\lambda_{i}}^{l*},{\lambda_{i}}^{g*},{\lambda_{i}}^{j*},{\lambda_{i}}^{r*}\right)$ in the relaxed three-layer Stackelberg game.

For the treatment of coupling resource constraints C1 and C5 in the relaxed problem, the existence of an equilibrium point means that there is a resource allocation scheme $\left\{{F_{i}}^{g*}\right\},\left\{{F_{i}}^{j*}\right\}$ such that, under the optimal pricing and offloading policy, these constraints are satisfied in the form of an equality (i.e., the resource is fully utilized), or the server consciously reserves part of the resource because it is not optimal to allocate all the resources. The essence of game equilibrium is that the competitive allocation of these scarce resources has reached a stable state.

To sum up, there is an equilibrium solution in the two-master-one-slave three-layer Stackelberg game model.

### Proof of uniqueness of equilibrium solution

Theorem 2. There is a unique Stackelberg equilibrium solution in the game model with relaxed strategy space (i.e. $Rol{e_{i}}^{j}\in \left[0,1\right]$).

Proof: We argue for the uniqueness of the equilibrium of the game after relaxation by showing that the optimal response of the players at each level is single-valued, given the strategies of the other players.

As the main leader of the three-tier game at the cloud edge, the utility of the cloud is defined as

$$U_{g}({M_{i}}^{g},{M_{j}}^{g},{F_{i}}^{g},J^{*},I^{*})=\gamma^{p}({P_{g}}^{comp}-{C_{g}}^{r})-\gamma^{t}{T_{i,g}}^{comp}\;=\sum_{i\in I}\gamma^{p}{M_{i}}^{g}{F_{i}}^{g}{S_{i}}^{g}-\sum_{i\in I}\sum_{j\in J}\gamma^{p}{M_{j}}^{g}D_{i}{\lambda_{i}}^{j}{S_{i}}^{j}-\sum_{i\in I}\gamma^{t}\frac{({\lambda_{i}}^{g}+{\lambda_{i}}^{r})\varphi_{i}}{{F_{i}}^{g}}.\;$$ (36)

Taking the partial derivative of the above equation, the result is as follows:

$$\frac{\partial U_{g}}{\partial {M_{i}}^{g}}=\gamma^{p}{F_{i}}^{g}{S_{i}}^{g}\geq 0,$$ (37)

$$\frac{\partial U_{g}}{\partial {M_{j}}^{g}}=-\gamma^{p}D_{i}{S_{i}}^{j}{\lambda_{i}}^{r}\leq 0,$$ (38)

$$\frac{\partial U_{g}}{\partial {F_{i}}^{g}}=\gamma^{p}{M_{i}}^{g}{S_{i}}^{g}+\frac{\gamma^{t}({\lambda_{i}}^{g}+{\lambda_{i}}^{r})\varphi_{i}}{{\left({F_{i}}^{g}\right)}^{2}}\geq 0.$$ (39)

Given the policies of the edge server and the mobile device, the partial derivative of the utility function *U*_*g*_ of the cloud server with respect to its decision variables ${M_{i}}^{g}$ and ${F_{i}}^{g}$ is always positive (see formulas (37) and (39)), so *U*_*g*_ is a strictly monotone increasing function with respect to ${M_{i}}^{g}$ and ${F_{i}}^{g}$. However, under the constraint of the coupling resource constraint C1, the cloud cannot allocate the maximum resource to all users without limit. The optimal resource allocation strategy must be an interior solution with tradeoffs among users, and the problem constitutes a convex optimization problem under linear constraints. Due to the strict monotonicity of the objective function and the convexity of the constraint set, this optimization problem has a unique optimal solution $\left({M_{i}}^{g*}{F_{i}}^{g*}\right)$. In the same way, for the pricing ${M_{j}}^{g}$, *U*_*g*_ is its monotonically decreasing function (formula (38)), and its minimum point on the interval $\left[\min{M_{j}}^{g},\max{M_{j}}^{g}\right]$ is unique.

As the co-leader of the three-tier game at the cloud edge, the utility of the edge server is defined as

$$U_{j}(G^{*},{m_{i}}^{j},{F_{i}}^{j},I^{*})=\beta^{p}({P_{j}}^{comp}+{P_{j}}^{relay})-\beta^{e}({E_{j}}^{comp}+{E_{j}}^{comm})-\beta^{t}{T_{i,g}}^{comp}\;\text{ =}\sum_{i\in I}\beta^{p}{m_{i}}^{j}{F_{i}}^{j}Rol{e_{i}}^{j}+\sum_{i\in I}\sum_{i\in J}\beta^{p}{M_{j}}^{g}D_{i}{\lambda_{i}}^{r}{S_{i}}^{j}\;\;-\;\sum_{i\in I}\beta^{e}k_{j}{\left({F_{i}}^{j}\right)}^{2}\varphi_{i}{\lambda_{i}}^{j}Rol{e_{i}}^{j}-\sum_{i\in I}{\sum_{i\in J}\beta^{e}P_{j}}^{g}\frac{D_{i}}{{R_{j}}^{g}}{\lambda_{i}}^{r}{S_{i}}^{j}\;\;-\sum_{i\in I}\beta^{t}\frac{{\lambda_{i}}^{j}\varphi_{i}}{{F_{i}}^{j}}.\;$$ (40)

Taking the partial derivative of the above equation, the result is as follows:

$$\frac{\partial U_{j}}{\partial {m_{i}}^{j}}=\beta^{p}{F_{i}}^{j}Rol{e_{i}}^{j}\geq 0,$$ (41)

$$\frac{\partial U_{j}}{\partial {F_{i}}^{j}}=\beta^{p}{m_{i}}^{j}Rol{e_{i}}^{j}+2\beta^{e}k_{j}{F_{i}}^{j}\varphi_{i}{\lambda_{i}}^{j}Rol{e_{i}}^{j}+\beta^{t}\frac{{\lambda_{i}}^{j}\varphi_{i}}{{\left({F_{i}}^{j}\right)}^{2}}\geq 0.$$ (42)

In the relaxed model, the role variable $Rol{e_{i}}^{j}$ of the edge server is continuous. Given the cloud strategy and terminal offloading decision, the utility function *U*_*j*_ is monotonically increasing with respect to the pricing ${m_{i}}^{j}$ (formula (41)), and the partial derivative with respect to the resource allocation ${F_{i}}^{j}$ is positive (formula (42)). Similar to the cloud, under the total resource constraint C5, the optimal solution of the edge server resource allocation is also a unique internal equilibrium point. Therefore, there is a unique optimal reaction triple $\left({m_{i}}^{j*},{F_{i}}^{j*},Rol{e_{i}}^{j*}\right)$ for the edge layer.

From the perspective of a follower, the utility of the mobile device is given by

$$U_{i}(G^{*},J^{*},{\lambda_{i}}^{l},{\lambda_{i}}^{g},{\lambda_{i}}^{j},{\lambda_{i}}^{r})=[{\lambda_{i}}^{l},{\lambda_{i}}^{g},{\lambda_{i}}^{j},{\lambda_{i}}^{r}]\cdot \left(-C_{i}\right),$$ (43)

where *C*_*i*_ is constant for $\lambda_{i}$, *U*_*i*_ can be simplified as

$$U_{i}(G^{*},J^{*},{\lambda_{i}}^{l},{\lambda_{i}}^{g},{\lambda_{i}}^{j},{\lambda_{i}}^{r})=C_{1}{\lambda_{i}}^{l}+C_{2}{\lambda_{i}}^{g}+C_{3}{\lambda_{i}}^{j}+C_{4}{\lambda_{i}}^{r}.$$ (44)

The utility function *U*_*i*_ of a mobile device is a linear function of its offload proportion $\lambda_{i}$ (formula (44)). Its maximization problem under constraints (C8 and C9) is a linear programming problem. The optimal solution of linear programming must exist in a certain pole of the feasible region, and among all the poles, the pole that maximizes the objective function is unique.

To sum up, in the relaxed game model, the optimal response maps of the players in each layer are single-valued. A Stackelberg game with a single-valued optimal response map has a unique equilibrium.

The above uniqueness conclusion holds for the relaxed game model. For the original problem containing the original binary constraints $Rol{e_{i}}^{j}\in \left\{0,1\right\}$(C7), its strategy space becomes a non-convex set due to discrete decisions. In this non-convex environment, the global uniqueness of the equilibrium point is difficult to be strictly guaranteed in theory, and there may be multiple local equilibria. Nonetheless, our proposed BI-PRO algorithm dynamically determines an optimal binary decision for the edge server role in each round (formula (9)) through its iterative mechanism. The results of a large number of simulation experiments show that the algorithm can stably converge to a high-performance system state in various scenarios, and the state shows good consistency under different initial conditions. This shows from the practical level that despite the theoretical possibility of multiple equilibria, the equilibrium solution found by the BI-PRO algorithm has significant practicality and robustness, and can be used as an effective and reliable solution to the complex optimization problem.

To sum up, the equilibrium point of the three-level Stackelberg non-cooperative leader-follower game is unique.

## Joint optimization algorithm based on backward regression

In this paper, we introduce a joint optimization algorithm for resource pricing, allocation, and computational offloading, termed BI-PRO, which is based on backward induction. This algorithm is designed for a three-level Stackelberg non-cooperative game. Central to the algorithm’s approach is an initial analysis of the follower’s (the mobile device’s) optimal response. Subsequently, the leader’s (the cloud server and the edge server) optimal strategy is derived. In detail, after the cloud and edge servers set their resource prices, the mobile device determines its offload proportion and associated decisions to maximize its utility. Conversely, anticipating the mobile device’s behavior, the cloud and edge servers adjust their resource pricing and allocation to optimize their own utilities. Through iterative rounds, both the leader and follower converge towards the Stackelberg equilibrium, a point where both strategies are at their optimum and neither party can unilaterally modify its strategy to achieve greater returns.

### Pseudocode and analysis

Since the core of the Stackelberg game lies in its hierarchical decision-making order, this algorithm operates by having the leader act first and the follower respond subsequently. The pseudocode for this algorithm is presented as Algorithm 1. Initially, the system’s simulation parameters and the maximum number of iterations, denoted as *E*, are set (line 1). Subsequently, the distances $\left\{{d_{i}}^{g},{d_{i}}^{j},{d_{j}}^{g}\right\}$ between the cloud, edge, and end devices are determined using the Euclidean formula (line 2). The transmission rates $\left\{{R_{i}}^{g},{R_{i}}^{j},{R_{j}}^{g}\right\}$ are then computed based on equations (6), (7), and (8) (line 3). Finally, the loop is iterated *E* times to calculate the Nash equilibrium optimal strategy of the three-level game (lines 4-34). In each iteration, for every cloud server *CS*_*g*_ (lines 5-19), an optimal resource allocation policy is initially determined. This involves specifying the frequency allocation amount of computing resources, represented as ${F_{i}}^{g}$ (line 6). Subsequently, based on the task offloading condition (where ${\lambda_{i}}^{g}\geq 0$ and ${S_{i}}^{g}=1$), a resource pricing strategy is conducted for tasks that are directly offloaded to it (lines 7-12). If the current pricing is within the allowable range after doubling (line 8), the pricing is doubled and the price floor is updated (line 9). Otherwise, record the price cap and recalculate the pricing using the dichotomy method (lines 10-11). The same pricing strategy also applies to the resource pricing ${M_{j}}^{g}$ with the edge server as the relay node (lines 13-18). For each edge server *ES*_*j*_ (lines 20-28), the resource allocation ${F_{i}}^{j}$ is calculated first (line 21), and according to the task offload condition (where ${\lambda_{i}}^{j}\geq 0$ and ${S_{i}}^{j}=1$ ), the task offloaded to it is priced (lines 22-27). The pricing strategy also adopts the dichotomy (lines 23-26). For each mobile device *MD*_*i*_ (lines 29-32), update the task offload proportions ${\lambda_{i}}^{l},{\lambda_{i}}^{g},{\lambda_{i}}^{j},{\lambda_{i}}^{r}$ according to the existence and uniqueness of the maximum utility of formula (30) based on the resource pricing and resource allocation at the cloud and edge ends (line 30). Then, the service selection states ${S_{i}}^{g},{S_{i}}^{j}$ are updated to be 0 or 1 according to the offload proportions ${\lambda_{i}}^{l},{\lambda_{i}}^{g},{\lambda_{i}}^{j},{\lambda_{i}}^{r}$ (line 31). Finally, when the iteration is completed, the utility values of the three parties, cloud, edge, and end, converge and stabilize, and the optimal resource pricing ${M_{i}}^{g},{M_{j}}^{g},{m_{i}}^{j}$, resource allocation ${F_{i}}^{g},{F_{i}}^{j}$ and task offloading ratio ${\lambda_{i}}^{l},{\lambda_{i}}^{g},{\lambda_{i}}^{j},{\lambda_{i}}^{r}$ are output (line 34).

**Algorithm 1 Resource pricing, allocation and task offloading algorithm based on reverse regression.**

**Input:** Set of locations $\left\{L_{g}\right\},\left\{L_{j}\right\},\left\{L_{i}\right\}$,Set of calculations $\left\{D_{i}\right\}$.

**Output:** ${M_{i}}^{g},{M_{j}}^{g},{m_{i}}^{j},{F_{i}}^{g},{F_{i}}^{j},{\lambda_{i}}^{l},{\lambda_{i}}^{g},{\lambda_{i}}^{j},{\lambda_{i}}^{r}$.

1: Initialize the simulation parameters of the system, the maximum number of iterations *E*.

2: Calculate the distance between cloud edge devices according to Euclid’s formula $\left\{{d_{i}}^{g},{d_{i}}^{j},{d_{j}}^{g}\right\}$.

3: The transmission rate $\left\{{R_{i}}^{g},{R_{i}}^{j},{R_{j}}^{g}\right\}$ is calculated according to formula (4-6), (4-7),(4-8).

4: **for** episode=1,…, *E* **do** //Performing Nash Equilibrium Iteration.

5:   **for** g=1,…,*G* **do** //The cloud server generates the optimal pricing strategy and resource allocation strategy.

6:   ${F_{i}}^{g}=({\lambda_{i}}^{g}+{\lambda_{i}}^{r})*D_{i}$

7:   **if** ${\lambda_{i}}^{g}\geq 0\text{ and }{S_{i}}^{g}==1$ **then**

8:    **if** *min* ${M_{i}}^{g}\leq 2{M_{i}}^{g}\leq \max$ ${M_{i}}^{g}$ **then**

9:     ${M_{i}}^{g}=2*{M_{i}}^{g};{M_{i,low}}^{g}={M_{i}}^{g}$

10:    **else**

11:     ${M_{i,high}}^{g}={M_{i}}^{g};{M_{i}}^{g}={M_{i,high}}^{g}+{M_{i,low}}^{g}/2$

12:    **end if**

13:    **if** ${\lambda_{i}}^{r}\geq 0\text{ and }{S_{i}}^{g}\text{==1}$ **then**

14:     **if** *min* ${M_{j}}^{g}\leq 2{M_{j}}^{g}\leq \max$ ${M_{j}}^{g}$ **then**

15:     ${M_{j}}^{g}=2*{M_{j}}^{g};{M_{j,low}}^{g}={M_{j}}^{g}$.

16:    **else**

17:     ${M_{j,high}}^{g}={M_{j}}^{g};{M_{j}}^{g}=({M_{j,high}}^{g}+{M_{j,low}}^{g})/2$

18:    **end if**

19:   **end for**

20:   **for** j=1,…,*J* **do** //The edge server generates the optimal pricing strategy and resource allocation strategy.

21:   ${F_{i}}^{j}={\lambda_{i}}^{j}*D_{i}$

22:   **if** ${\lambda_{i}}^{j}\geq 0\text{ and }{S_{i}}^{j}==1$ **then**

23:    **if** *min* ${m_{i}}^{j}\leq 2{m_{i}}^{j}\leq \max$ ${m_{i}}^{j}$ **then**

24:     ${m_{i}}^{j}=2{m_{i}}^{j};{m_{i,low}}^{j}={m_{i}}^{j}$

25:    **else**

26:     ${m_{i,high}}^{j}={m_{i}}^{j};{m_{i}}^{j}=({m_{i,high}}^{j}+{m_{i,low}}^{j})/2$

27:    **end if**

28:   **end for**

29:   **for** i=1,…,*I* **do** //Mobile device generates optimal task offloading strategy

30:   Update the task offload proportion ${\lambda_{i}}^{l},{\lambda_{i}}^{g},{\lambda_{i}}^{j},{\lambda_{i}}^{r}$ according to the existence and uniqueness of the maximum utility of formula (30).

31:   Update ${S_{i}}^{g},{S_{i}}^{j}$ according to ${\lambda_{i}}^{l},{\lambda_{i}}^{g},{\lambda_{i}}^{j},{\lambda_{i}}^{r}$

32:   **end for**

33: **end for**

34: **return** ${M_{i}}^{g},{M_{j}}^{g},{m_{i}}^{j},{F_{i}}^{g},{F_{i}}^{j},{\lambda_{i}}^{l},{\lambda_{i}}^{g},{\lambda_{i}}^{j},{\lambda_{i}}^{r}$

### Complexity analysis

During the execution of the Nash equilibrium iteration process, O(*E*) loops need to be performed. In each loop, first, each cloud server (*G* servers) updates its resource pricing and allocation strategy. Each cloud server performs a binary search to update resource prices for associated devices, with a time complexity of O(*G*log*M*), where M represents the search range for resource pricing in cloud servers. Subsequently, each edge server (*J* servers) updates its resource pricing and allocation strategy. Similarly, each edge server also performs a binary search to update pricing, resulting in a complexity of O(*J*log*m*) for this phase, where m denotes the search range for resource pricing in edge servers. Finally, each mobile device (*I* devices) efficiently updates its task offloading decision according to the closed-form solution (Formula 30), with a time complexity of O(*I*). Comprehensive analysis shows that the total time complexity per iteration is O(*G*log*M*+*J*log*m*+*I*), while the overall algorithmic time complexity reaches O(*E*(*G*log*M*+*J*log*m*+*I*)). Given that *E*, *G*, and *J* are small constants, *I* remains bounded, and both *M* and *m* have limited magnitudes, the total computational overhead of Algorithm 1 is acceptable.

### Convergence analysis

In order to ensure the effectiveness of the proposed BI-PRO algorithm, its convergence is strictly analyzed in this section. The core of the algorithm is an iterative process on the strategy space of cloud, edge and end, which can be defined as a mapping $x^{\left(k+1\right)}=\phi (x^{\left(k\right)})$, where $x=({M_{i}}^{g},{F_{i}}^{g},{m_{i}}^{j},{F_{i}}^{j},\lambda_{i})$ is the joint strategy vector.

Theorem 3. The sequence $\left\{x^{\left(k\right)}\right\}$ generated by the BI-PRO algorithm converges to a Stackelberg equilibrium point.

Proof: The convergence is guaranteed by the synergy of the various parts of Algorithm 1.

Strong convergence of pricing update: The resource pricing strategy of cloud servers and edge servers (lines 7-18, 22-27) adopts the binary search method. The method itself constitutes a contraction mapping with a contraction factor of *L*_*p*_ = 1/2, which ensures that the pricing strategy can quickly and monotonically approach the optimal value.

Smoothness of resource and offload decisions: The resource allocation ${F_{i}}^{g},{F_{i}}^{j}$ is directly linearly determined by the amount of task offload. The mobile’s task offload decision $\lambda_{i}$ (line 30) is a continuous function of resource pricing and allocation. The update process of these parts is continuous and smooth.

The mapping ϕ is a composite of the above pricing compression mapping and the resource unloading continuous mapping. According to Banach’s fixed point theorem, a compound mapping on a compact strategy space can globally converge to a unique fixed point if its key part (pricing update) is contractive and its other parts are continuous.

The following simulation results verify the theoretical analysis: in all test scenarios, the total utility of the system and the utility of each participant can reach a stable state within a limited number of iterations, which confirms the robustness and convergence of the algorithm.

## Simulation results and analysis

In this paper, we utilize Matlab software to develop the simulation environment and conduct subsequent experiments. Initially, the parameters of the simulation environment are configured based on real-world application scenarios. Our findings confirm that, through a specified number of iterations, the maximum payoff for each participant in the game can stabilize, indicating that the proposed three-layer game model from cloud to edge to end achieves equilibrium. In the concluding phase, we compare the performance of our proposed BI-PRO with the All-local and Random approaches, further underscoring the superiority of BI-PRO.

### Simulation parameter setting

The simulation experiment, set within a cloud-edge-end framework, incorporates 2 cloud servers, 6 edge servers, and 9 mobile devices. The site dimensions span an area of 1000 x 1000 *m*^2^. The locations of the two cloud servers are set as (250 m, 500 m, 0 m) and (750 m, 500 m, 0 m). The locations of the 6 edge servers are set as: (350 m, 350 m, 50 m), (350 m, 600 m, 50 m), (350 m, 850 m, 50 m), (850 m, 350 m, 50 m), (600 m, 850 m, 50 m) and (850 m, 850 m, 50 m). The locations of the mobile units are: (75 m, 739 m, 0 m), (132 m, 517 m, 0 m), (263 m, 693 m, 0 m), (395 m, 437 m, 0 m), (574 m, 161 m, 0 m), (562 m, 663 m, 0 m), (573 m, 631 m, 0 m), (639 m, 915 m, 0 m) and (893 m, 779 m, 0 m). Calculate the distance between the cloud server, the edge server, and the mobile device according to the Euclidean distance formula. The set of task data randomly generated by the mobile device is 849 MB, 613 MB, 539 MB, 463 MB, 406 MB, 621 MB, 550 MB, 419 MB, 620 MB. The computing frequency of the cloud server is *F*^*g*^ = 24 GHz, and the computing frequency of the edge server is *F*^*j*^ = 3 GHz. The settings of other parameters in the simulation experiment are shown in Table 3.

**Table 3:** Simulation parameter setting.

Parameter	Numerical value	Parameter	Numerical value
$B_{i}^{g}$	2 MHz	$N_{NLos}$	30 dB
$B_{i}^{g}$	2 MHz	*k*_*j*_	10^−27^
$B_{j}^{g}$	20 MHz	*k*_*l*_	10^−26^
$P_{i}^{g}$	5 W	$\gamma^{p},\gamma^{t}$	(0.8,0.2)
$P_{i}^{j}$	5 W	$\beta^{p},\beta^{e},\beta^{t}$	(0.4,0.4,0.2)
$P_{j}^{g}$	12 W	$\alpha_{l}^{\text{time}},\alpha_{l}^{\text{energy}}$	(0.3,0.7)
$\sigma^{2}$	-100 dBm	$\alpha_{ig}^{\text{pay}},\alpha_{ig}^{\text{time}},\alpha_{ig}^{\text{energy}}$	(0.3,0.2,0.5)
$\eta_{0}$	0.001	$\alpha_{ij}^{\text{pay}},\alpha_{ij}^{\text{time}},\alpha_{ij}^{\text{energy}}$	(0.3,0.2,0.5)
$\varphi_{i}$	1500Megacycles	$\alpha_{r}^{\text{pay}},\alpha_{r}^{\text{time}},\alpha_{r}^{\text{energy}}$	(0.3,0.2,0.5)

### Analysis of experimental results

In the conducted experiment, when the number of iterations, denoted as *E*, reaches 300—marking stable convergence—the task offloading policies across all mobile devices are delineated in Table 4. The data reveals that the majority of computational tasks from mobile devices are directly offloaded to the edge server and executed locally. Notably, the volume of tasks processed by the edge server significantly surpasses that of local computing. A limited number of mobile devices have their computational tasks handled both on the cloud server and locally; this includes instances where the edge server functions as a relay, as exemplified by mobile device 2.

**Table 4:** Simulation parameter setting.

Mobile device	Clouds(%)	Edge layer(%)	Local layer(%)
1	0	96.2	3.8
2	94.8	(94.8)	5.2
3	0	100	0
4	0	93.1	6.9
5	92.2	0	7.8
6	0	94.6	5.4
7	0	94.1	5.9
8	0	92.5	7.5
9	0	94.9	5.1

**Fig 2:**
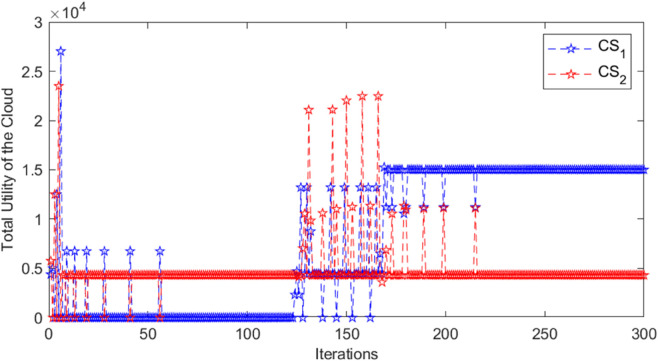
Total cloud utility for different iterations.

**Fig 3:**
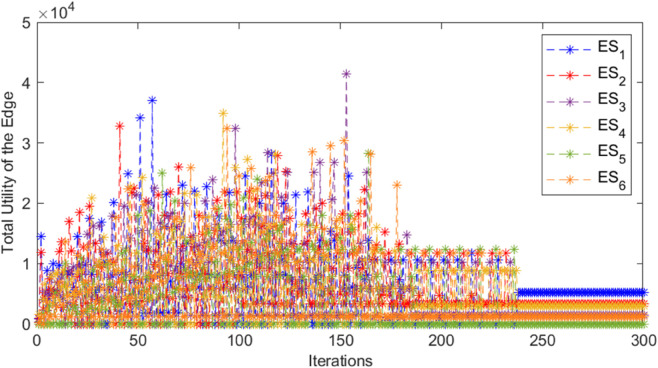
Total utility of edge end for different iterations.

**Fig 4:**
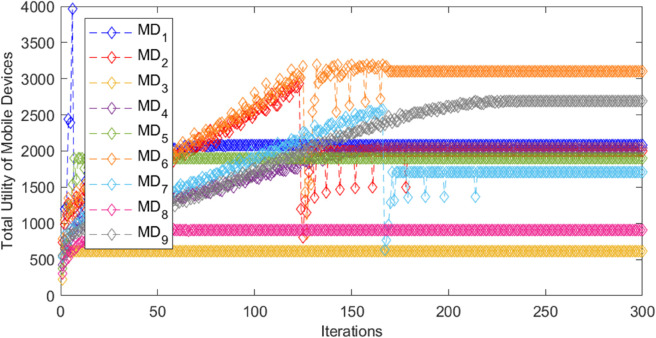
Total utility of mobile devices for different iterations.

In the three-tiered game, the cloud, edge, and mobile device collectively achieve the maximum total revenue and stabilize the iterative process, as shown in Figs 2, 3, and 4. In Fig 2, when the cloud server *CS*_1_ has 120 iterations, the total revenue suddenly and unstably increases or decreases alternately, and reaches the maximum value and tends to be stable when it is nearly 160 iterations. The total revenue of cloud server *CS*_2_ stabilizes around 220 iterations. In Fig 3, the total return of the marginal end exhibits significant fluctuations before achieving stability, suggesting an intense game process. After about 240 iterations, each edge tends to be stable and reaches the equilibrium state of the game. In Fig 4, when *MD*_2_ is iterated to nearly 120 times, the cost suddenly changes dramatically, and it does not stabilize until 180 times, indicating that the switching of unloaded objects has occurred, resulting in an impact on revenue. Similarly, the cost of *MD*_7_ also experienced large fluctuations, and gradually stabilized after about 160 iterations. The cost of other mobile devices changes gently, and most of them converge to a stable state quickly, that is, the game equilibrium state, indicating that the tasks on them seldom switch offloading objects. In summary, after a specified number of iterations, the payoffs or costs of all game players converge to a consistent value, thereby achieving the game equilibrium solution. When the aggregate payoff or cost for the cloud, edge, and mobile devices reaches stability, the overall system’s payoff is also optimized.

**Fig 5:**
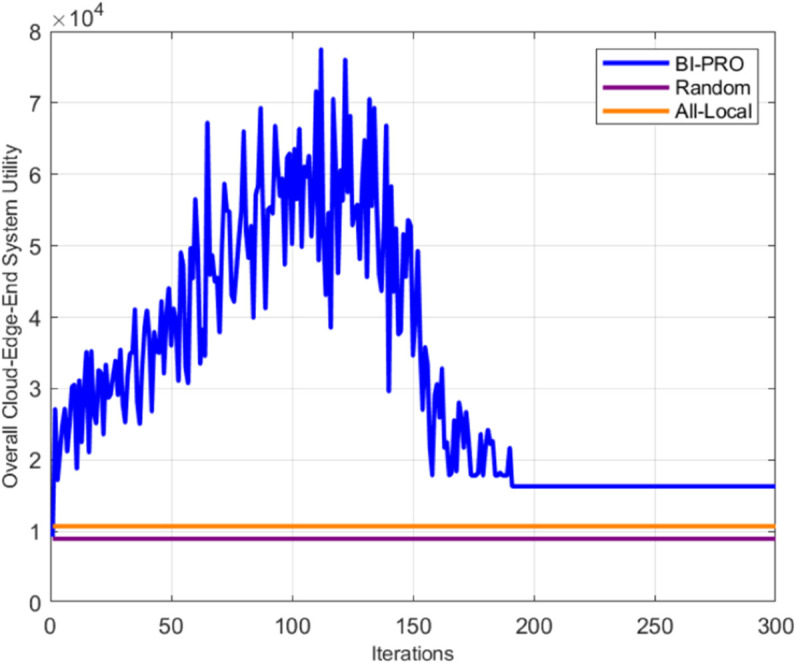
Total utility of the system for different iterations.

In Fig 5, we evaluate the total system revenue of three algorithms: the proposed BI-PRO, the random offload (Random), and the all-local offload (All-Local) across various iterations. It is evident that after approximately 190 iterations, the BI-PRO algorithm’s total revenue approaches stability, indicating convergence to its game equilibrium state. In contrast, the system revenues for both the Random and All-Local algorithms remain constant regardless of the iteration count. Notably, the BI-PRO algorithm consistently outperforms the other two, demonstrating its efficacy and superiority in achieving an optimal unloading strategy at equilibrium.

**Fig 6:**
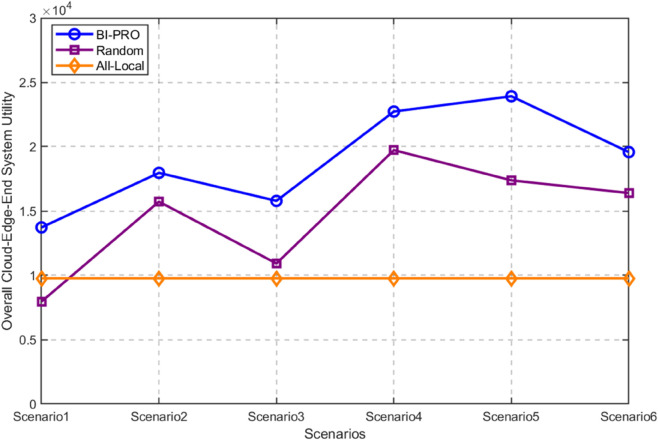
Total system utility for different mobile device locations.

In various scenarios, the coordinates of mobile devices are randomly produced within the confines of the site. We compared the total system revenue of the three algorithms, as shown in Fig 6. When the mobile devices are in different locations, the BI-PRO algorithm always outperforms the Random algorithm and the All-local algorithm, and the Random algorithm is better than All-local algorithm as a whole. The aforementioned phenomenon demonstrates that the algorithm’s total revenue remains unaffected by the geographical distribution of mobile devices, showcasing its superior and stable performance.

**Fig 7:**
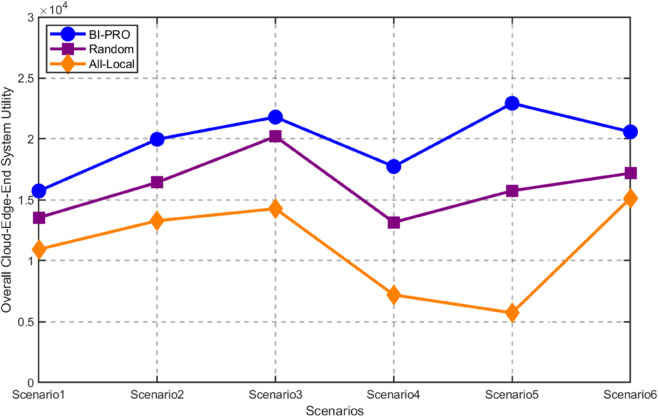
Total system utility for different task data volumes.

The size of the task data for all mobile devices is randomly generated between 200 MB and 1GB [34], and we compare the total system revenue of the three algorithms. As shown in Fig 7, regardless of the quantity of task data, the overall system revenue generated by the BI-PRO algorithm consistently outperforms that of both the Random and the All-local algorithms. Notably, while variations in the size of the mobile device’s task data do influence cost, thereby causing fluctuations in the total system revenue, the proposed BI-PRO algorithm remains demonstrably superior in performance.

**Fig 8:**
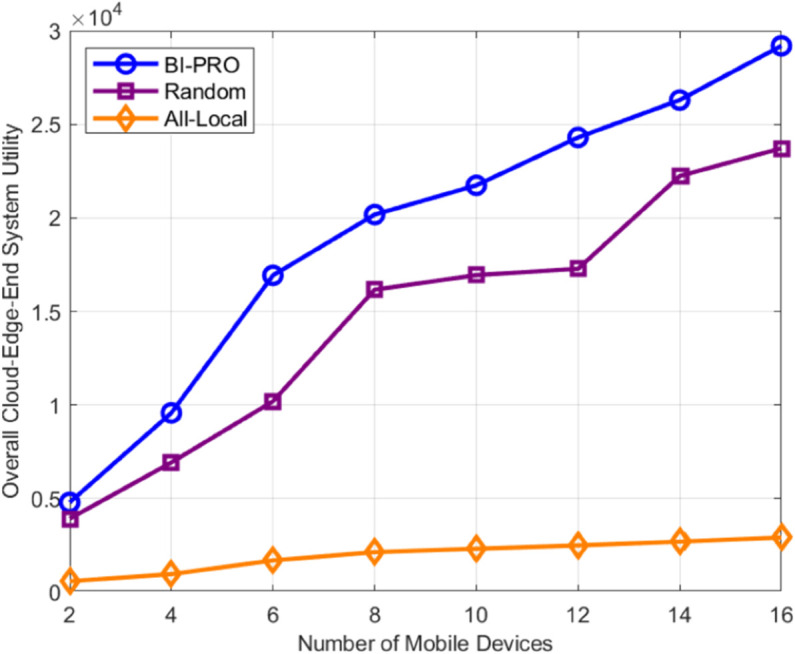
Total system utility for different number of mobile devices.

As illustrated in Fig 8, we compare the total system revenue of three algorithms at equilibrium, under varying numbers of mobile devices. It is evident that as the number of mobile devices increases, so does the total revenue for all three systems. Notably, the BI-PRO algorithm consistently outperforms both the random and all-local algorithms. Therefore, for large-scale mobile device scenarios, the BI-PRO algorithm has a certain potential performance advantage, that is, it has good scalability.

## Conclusion

This paper presents a joint optimization method for computing offloading, resource allocation, and pricing within a cloud-edge-end collaboration scenario. The proposed method is based on the Stackelberg game and aims to maximize system utility by considering latency, energy consumption, and revenue. The study first establishes a Stackelberg game model comprising cloud servers, edge servers, and mobile terminal devices. In this model, the cloud server dominates resource pricing, the edge server oversees resource allocation, and the mobile terminal manages computing offloading. Then, the equilibrium analysis of this game model is conducted and it is proved that there exists a unique equilibrium point. Next, a backward induction-based resource pricing, resource allocation, and computing offload optimization algorithm is proposed, BI-PRO. Finally, the effectiveness of the proposed BI-PRO algorithm is verified through simulation experiments. Compared with other benchmark methods, this algorithm reaches the equilibrium state after multiple iterations, can perform task offloading and resource allocation more optimally, and can achieve higher system revenue in various scenarios. Although the game theory method performs well in collaborative optimization problems, it still needs to be improved in high-dimensional dynamic environments. Future work will introduce data-driven methods (such as deep reinforcement learning) to achieve adaptive computing offloading and combine federated learning to achieve cross-node collaboration under privacy protection.
